# Cervicogenic headache alleviation after cervical coblation nucleoplasty: A prospective cohort study

**DOI:** 10.1097/MD.0000000000004786

**Published:** 2016-09-30

**Authors:** Liangliang He, Jianning Yue, Liqiang Yang, Baishan Wu, Guoqing Cao, Yuna Guo, Guanghui Lai, Yuanzhang Tang, Jiaxiang Ni

**Affiliations:** Department of Pain Management, Xuanwu Hospital, Capital Medical University, Xicheng, Beijing, China.

**Keywords:** cervical disc herniation, cervical surgery, cervicogenic headache, nucleoplasty

## Abstract

A degenerative cervical disc is a pain generator for headaches, and headaches can benefit from cervical prolapse surgery. However, as an alternative intervention for open cervical surgery, no study has reported whether headaches can benefit from cervical nucleoplasty.

The objective of this study was to evaluate the efficacy of cervical coblation nucleoplasty in the treatment of cervicogenic headaches.

In a prospective cohort study performed between December 2013 and August 2015, 20 patients with cervicogenic headaches undergoing cervical nucleoplasty for shoulder-arm pain were recruited into group C, and 20 patients with cervicogenic headaches undergoing lumbar nucleoplasty for low back pain, matched for age and sex, were recruited into group L. Cervicogenic pain was diagnosed according to the International Headache Society criteria. During the 24-month follow-up, pain visual analog scale (VAS) scores were collected as the primary outcomes, and significant pain relief rate, Neck Disability Index (NDI) headache scores, and Patients Satisfaction Index (PSI) scores were recorded as secondary outcomes to evaluate headache severity and physical function postoperatively.

During the 24-month follow-up, a significant decrease in headache VAS scores was observed in group C but not in group L. NDI and PSI scores in group C were better than those in group L. In comparison with the final follow-up, no significant differences in the NDI and PSI scores were found in all observations after surgery. In comparison to group L, ≥50% pain relief was significantly better in group C. No serious complications were observed except for ≤20% of ecchymoma at the needle insertion site.

This prospective study indicated that cervicogenic headaches may benefit from nucleoplasty.

## 1 Introduction

The cervicogenic headache (CEH) is characterized by unilateral headache symptoms arising from the neck that radiate to the frontal-temporal and possibly to the supraorbital region.^[1]^ According to the criteria of Sjaastad, the prevalence of CEHs is 1%^[2]^; according to the criteria of the International Headache Society (IHS), the prevalence is between 0.17% and 2.5%^[3,4]^; however, according to the criteria of the Cervicogenic Headache International Study Group (CHISG), the prevalence is high at 4.1%.^[5]^ CEHs have a tremendous impact on physical and mental health and seriously affect the quality of life.

Since the concept of the CEH was presented at the first World Congress of Headache in 1983,^[6]^ the view of the neck as a pain generator for headaches has gained wide acceptance.^[7,8]^ Based on this view, an appropriate therapeutic method directed at cervical nociceptive structures offers benefit for CEHs. After evaluating the upper cervical nerve root and facet joints,^[9,10]^ the role of degenerative cervical discs in CEHs was noticed.^[11,12]^ Since surprising headache relief after standard neck surgery for myelopathy or radicular shoulder-arm syndrome was previously reported,^[11,12]^ a positive association between CEHs and open cervical surgery has been indicated.^[13–15]^

In contrast to adherence to stepped care programs, a minimally invasive intervention, especially a coblation nucleoplasty, was recommended to bridge unresponsive conservative therapy and open spine surgery.^[16]^ To date, only 1 case of significant headache relief after coblation nucleoplasty in C6–7 was reported in 2004,^[17]^ and up to 6 months of 100% headache relief fulfilled Criterion D of the IHS classification system for CEH as follows: the pain resolves within 3 months after treatment of the neck.^[18]^ However, the connection between the CEH and cervical nucleoplasty has no powerful evidence. Therefore, we hypothesized that CEH can benefit from cervical nucleoplasty in a prospective cohort study.

## 2 Methods

This prospective cohort study was performed after obtaining approval from the Institution's Ethics Examining Committee of Human Research. Between December 2013 and August 2015, 20 patients with CEHs undergoing cervical nucleoplasty for shoulder-arm pain were recruited for the therapy group (group C), and 20 patients with CEHs undergoing lumbar nucleoplasty for back-leg pain were recruited for the control group (group L).

The inclusion criteria were as follows: met the inclusion criteria of cervical or lumbar nucleoplasty (Table 1); met IHS's diagnostic criteria for CEH (Table 2); and experienced CEHs with a short-term response or a lack of response to conservative treatment (medication, physical or manual therapy, etc.), trigger point injection or nerve block injection therapies (nerve occipitals major/minor, medial branch or epidural injection, etc.).

**Table 1:**
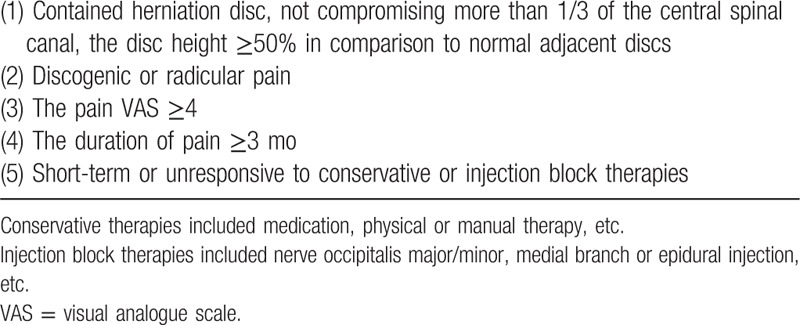
Inclusion criteria of nucleoplasty.

**Table 2:**
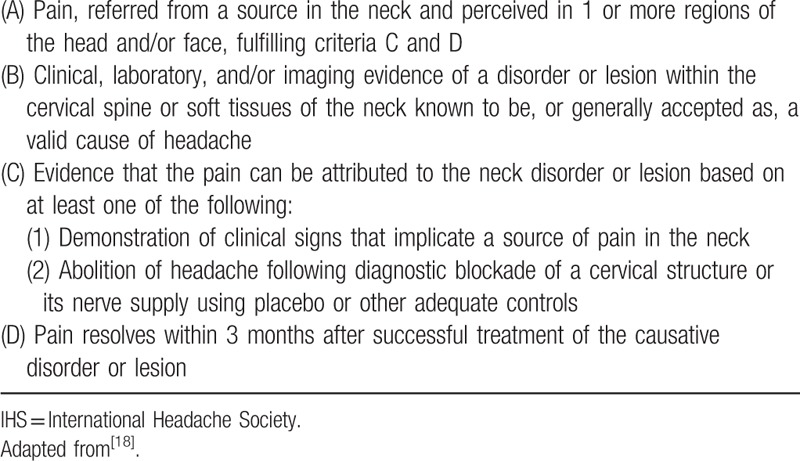
Diagnostic criteria for cervicogenic headache according to IHS^[18]^.

Patients with whiplash injury, post-trauma headache, an organic disease of the brain, disc herniation with sequestration, infection or spinal instability, spinal fractures, tumor, coagulopathy, or receiving psychological or psychiatric therapy were excluded.

All procedures were performed under fluoroscopic guidance with anterior–posterior and lateral views in an operating room using a sterile technique. Patients in group C were placed in the supine position and received a puncture in a left or right anterior approach, and patients in group L were placed in a lateral position and received puncture in a left or right posterolateral approach. In group C, the coblation wand (UNITEC, China America United Technology Co. Ltd, Beijing, China) was inserted into the opposite posterior annulus/nucleus junction of the cervical disc, and, in group L, the coblation wand was inserted into the posterior annulus/nucleus junction of the lumbar disc. Once the position of the wand tip was confirmed, the cervical nucleoplasty was conducted by rotating the wand 360°, and lumbar nucleoplasty was accomplished by creating six channels in the annulus at the 12, 2, 4, 6, 8, and 10 o’clock positions with the radio-frequency controller set to an intensity of 2′. Of note, coagulation should be performed to verify the presence of movement or paresthesia in the patient's upper or lower limbs before nucleoplasty.

After the nucleoplasty, all patients were advised to avoid long-term lowering of the head and protect cervical health. Clinical outcomes were assessed at 1 week and 1, 3, 6, 12, 18, and 24 months postoperatively. In this study, the clinical outcomes only focused on headache; therefore, the data regarding discogenic or radicular pain in the neck, shoulder, and arm were not included.

Headache severity, as the primary outcome, was measured with the visual analog scale (VAS) of pain. The secondary outcomes included the degree of significant pain relief (postoperative pain relief ≥50% compared with the preoperative state), the Neck Disability Index (NDI) headache score (0 = I have no headache at all; 1 = I have slight headaches that come infrequently; 2 = I have moderate headaches that come infrequently; 3 = I have moderate headaches that come frequently; 4 = I have severe headaches that come frequently; and 5 = I have headaches almost all the time), and the Patient Satisfaction Index (PSI) score (1 = surgery met my expectation; 2 = I did not improve as much as I had hoped, but I would undergo the same operation for the same results; 3 = surgery helped, but I would not undergo the same operation for the same outcome; and 4 = I am the same or worse compared to before surgery).

Twenty patients were enrolled in a pilot study with 10 patients in each group, and the 6-month intermediate step of analysis demonstrated that postoperative pain relief ≥50% was reported in 70% (7/10) of patients who underwent cervical nucleoplasty and only 10% (1/10) of patients in the control group. According to the sample size calculation by the incidence of an effective treatment from 10% to 70% with α of 0.05 and β of 0.1 (power 90%), 12 patients were required for each group. In consideration of dropouts and censorings, 20 patients were recruited for each group.

All data were processed by SPSS software version 19.0, and statistical significance was declared at the level of *P* ≤ 0.05 (2-tailed). Normally distributed continuous data on patient demographics and pain characteristics were reported as the mean ± standard deviation and calculated using independent samples *t*-tests, and categorical data (gender) were analyzed with the chi-squared test between the 2 groups. A generalized linear mixed model (GLMM) was performed to evaluate the changes in pain VAS data for the repeated measurements with missing values. A generalized estimating equations model (GEE) was used to investigate the significance of ranked ordinal data (NDI and PSI) in the repeated measures design. Kaplan–Meier survival analysis was using to estimate whether the pain relief (postoperative pain relief ≥50% compared with baseline) was performed identically in the study and control groups.

## 3 Results

There were no differences in patients’ basic characteristics, such as age, weight, height, and pain duration, between the study and the control groups (Table 3).

**Table 3:**
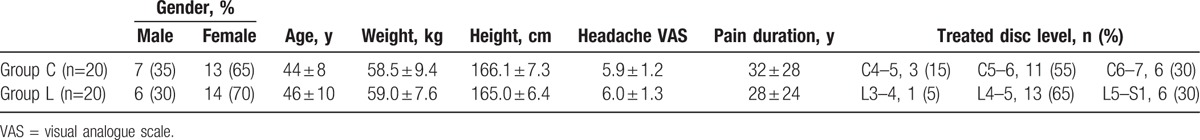
Data of demographic characteristics, preoperative headache VAS, pain duration, and treated disc level (mean ± standard).

### 3.1 VAS

**Figure 1:**
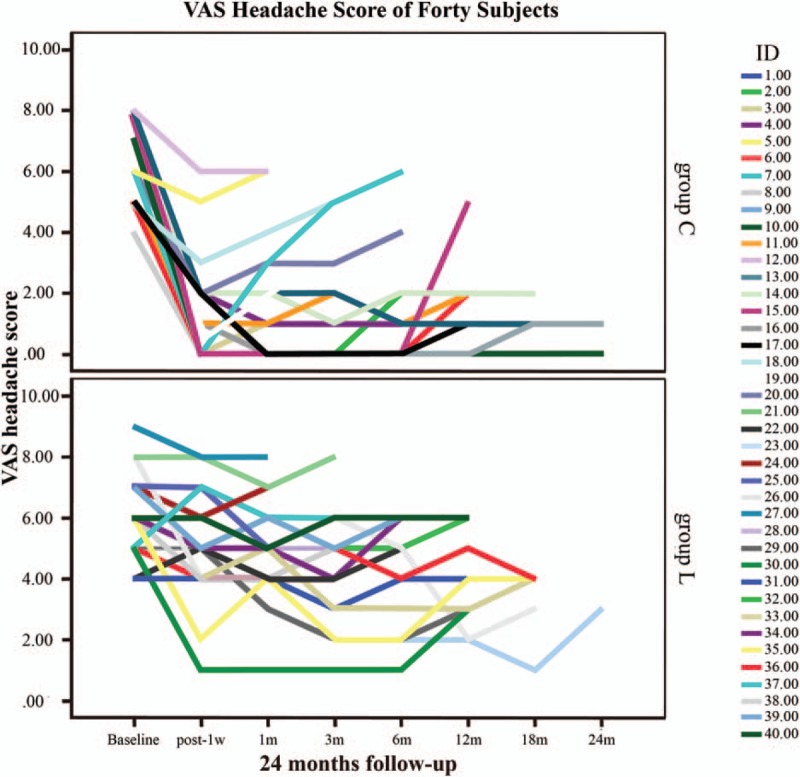
Forty patients’ headache visual analogue scale scores with missing values in the 2 groups during the 24-month follow-up period.

A total of 40 patients’ pain VAS scores with missing values are shown in Fig. 1. Six patients in group C and 12 patients in group L received injection therapy due to unbearable pain; complete data were obtained in 22 patients.

**Figure 2:**
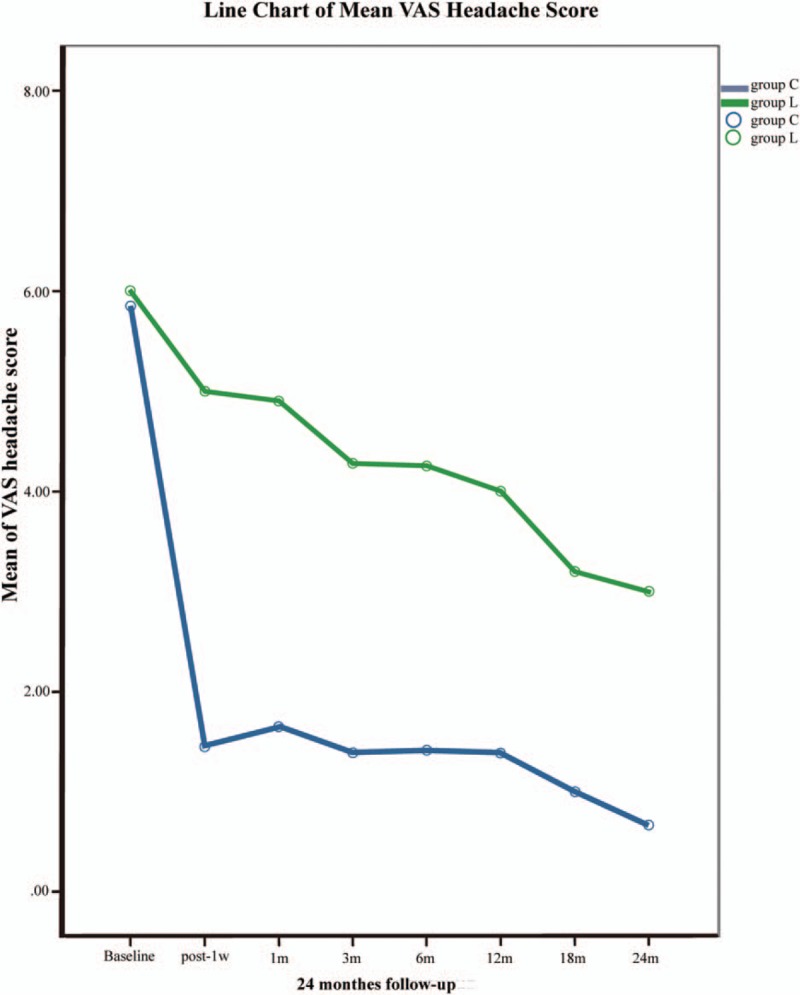
Changes in the patients’ visual analogue scale scores in the 2 groups during the 24-month follow-up period.

The VAS scores were approximately the same among the 2 groups at baseline (5.9 ± 1.2 in group C, 6.0 ± 1.3 in group L) (Fig. 2, Table 4). One week after surgery, the VAS scores of patients in group C rapidly declined compared to group L, and significant differences were observed (1.5 ± 1.7 in group C, 5.0 ± 1.8 in group L). Although not statistically significant, it was observed that the VAS scores of the treatment group exhibited a tendency to slowly decline and were significantly different from those of group L throughout the duration of the subsequent follow-up observations (Fig. 2, Table 4).

**Table:**
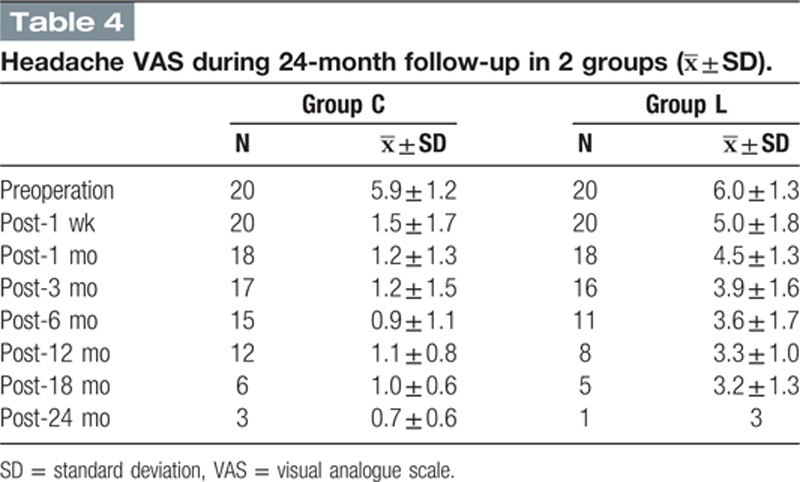


A GLMM was used to study the relationship between the treatment and the patients’ VAS score changes. Group effect (fixed), time effect (fixed), interaction effect of group and time (fixed), as well as subject effect (random), and interaction effect of subject and time (random) were assessed in the mixed models. The result of the GLMM indicated that both the group factor (treatment factor, F = 8.864, *P* = 0.004 and <0.05) and the time factor (F = 50.265, *P* = 0.000 and <0.05) had a significant effect on the pain VAS scores. Moreover, a significant interaction effect of group and time factors was found, which indicated that the speed of the VAS decline was significantly different (F = 4.778, *P* = 0.03 and >0.05). After the estimation of the fixed-effects parameters, the group factor was considered a significant predictor of VAS scores (*P* = 0.004 and <0.05). The average VAS value of group L was predicted to be 5.839697 compared with 4.162136 in group C. The estimated average VAS value decline velocity based on the slope of the VAS line was 0.270379 in group L and 0.511425 in group C. One could draw a conclusion that VAS scores declined more rapidly in group C after the surgery, which agreed with the VAS line chart.

The model has been verified as valid and feasible with a covariance parameter test (*P* = 0.000 and <0.05).

### 3.2 NDI and PSI

GEEs were employed to analyze the ranked ordinal data (NDI and PSI) for the repeated measurements. Our results showed that there were significant differences in NDI and PSI between the 2 groups (Wald Χ^2^ = 28.983, *P* = 0.000 and <0.05, and Wald Χ^2^ = 33.055, *P* = 0.000 and <0.05, respectively). Moreover, the parameter estimates were more accurate with the odds ratio (OR) = e^3.345^ = 28.361, 95% confidence interval (CI) (e^2.126^, e^4.563^) = (8.381, 95.871). This analysis revealed that the NDI in group C was better than that in group L. In comparison with the final follow-up, no significant difference in NDI was found in all observations after surgery. PSI had a similar result to NDI between the 2 groups with OR = e^3.933^ = 51.060, 95% CI = (e^2.592^, e^5.273^) = (13.356, 195.000).

### 3.3 Pain relief ≥50%

**Figure 3:**
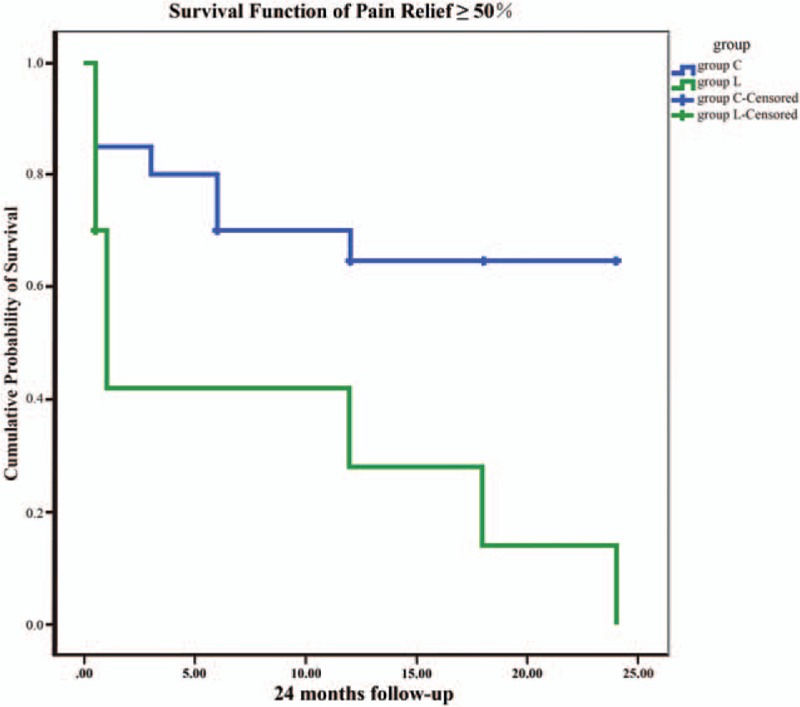
Kaplan–Meier survival curve to depict pain relief throughout the 24-month follow-up period.

To describe pain relief throughout the follow-up period, Kaplan–Meier survival analysis was applied as an effective analytic method. The Kaplan–Meier survival curve of 50% pain relief (Fig. 3) was significantly better in group C compared with group L, which is coincident with the results of the survival analysis function (*P* = 0.08 and <0.05).

### 3.4 Complications

Four (20%) patients in group C and 2 (10%) patients in group L experienced ecchymoma at the needle insertion site, and 5 (25%) patients in group C and 6 (30%) patients in group L complained of soreness, but the symptoms completely disappeared 2 weeks after the operation. No significant difference was found between the 2 groups.

## 4 Discussion

The clinical data in this study showed a significant decrease in pain VAS scores in patients with CEHs after cervical nucleoplasty, but no similar tendency was observed after lumbar nucleoplasty over a 12-month follow-up.

The CEH is perceived pain in the head, but the pain generator is the cervical spine.^[7,8]^ According to IHS diagnostic criteria for CEHs, 67% (8/12) of patients reported headache improvement or disappearance after surgery with removal of the cervical disc prolapse at the 3-month follow-up in a prospective, controlled study in 2007.^[13]^ According to the diagnostic criteria of CHISG for CEH, 63% (20/32) of patients with unilateral CEHs and 64% (18/28) of patients with bilateral CEHs experienced a mean of 19.8 and 25.5 months, respectively, of pain freedom or improvement (>50%) after an anterior cervical discectomy in 2008,^[14]^ and VAS pain scores decreased from 8.1 preoperatively to 3.1 postoperatively after anterior cervical decompression and fusion at the conclusion of a 12-month follow-up in a retrospective study of 33 patients in 2015.^[15]^ The optimistic therapeutic effect indicated that CEH can benefit from open cervical surgery for degenerative cervical disc disease.

Cervical nucleoplasty, as an alternative to open spine surgery, is one effective minimally invasive intervention for degenerative cervical disc disease by removing volume from the nucleus, reducing the intradiscal pressure, altering the intradiscal biochemical status, and ablating nociceptors in the innervated disc.^[19]^ However, this leads to the question of whether CEH can benefit from nucleoplasty.

In 2004, a case report of 100% relief in headaches after cervical nucleoplasty was published.^[17]^ Although up to 6 months of pain relief fulfilled diagnostic criteria “D” of IHS for CEH, the evidence level is limited. Our study found that VAS headache scores decreased from 5.9 ± 1.2 at baseline to 1.5 ± 1.7 one week after cervical nucleoplasty, but no significant reduction was detected in mean VAS headache scores from 6.0 ± 1.3 at baseline to 5.0 ± 1.8 after lumbar nucleoplasty. During the 24-month follow-up, a significant descending tendency in headache VAS scores was observed after cervical nucleoplasty but not after lumbar nucleoplasty, which strengthens the evidence to support that patients with CEHs may benefit from nucleoplasty.

Similar to a previously published report by Schrot et al,^[20]^ the NDI headache score was used to evaluate headache beyond the frequency and severity of symptoms in our study. In addition, in line with Liu's study,^[15]^ PSI was used to assess patient satisfaction with the outcome of nucleoplasty. After anterior open cervical surgery, only 13% to 17% of patients reported NDI headache scores of ≥3 in Schrot's study^[20]^ and 88% of patients reported PSI scores of ≤2 in Liu's study.^[15]^ In this study, poor results were shown on NDI headache scores and PSI scores after lumbar nucleoplasty, but better results were evident after cervical nucleoplasty. As an alternative to open surgery, cervical nucleoplasty can produce similar therapeutic efficacy in patients with CEH.

In this study, 3/20 patients underwent C4–5 nucleoplasty, 11/20 underwent C5–6 nucleoplasty, and 6/20 underwent C6–7 nucleoplasty, which is in accordance with the idea that lower cervical discs play an important role in the occurrence of CEH.^[13]^ According to the distribution mapping of evoked headache by lower cervical discography, Schellhas et al^[21]^ reported that C4–5 cervical discs evoked headache in the area of the mastoid, temporomandibular joint, occiput, parietal and craniovertebral junction, C5–6 cervical discs evoked headache in the occiput and craniovertebral junction, and Splipman et al^[22]^ reported that C4–5, C5–6, and C6–7 cervical discs evoked headache in the suboccipital and occipital areas. According to the postoperative outcomes of headache relief after lower cervical open surgery, Diener et al^[13]^ reported that cervical disc prolapse below the level of C4 was associated with CEH in the first prospective study, Schrot reported significant headache relief after open surgery at C4–5 in 13 patients, C5–6 in 146 patients, and C6–7 in 96 patients out of a total of 260 patients,^[20]^ and Jansen et al^[14,23]^ reported that some patients with CEH may benefit from surgical intervention mainly on a lower cervical disc. Therefore, the positive association between lower cervical disc and headache was identified by cervical discography and cervical open surgery.

Although the pathological mechanism of lower cervical disc involvement in CEH is unclear, the positive outcomes in this study are possibly due to the following: nucleoplasty resulted in decompression of the lower cervical nerve root^[19]^ because pain afferents from the lower cervical root possibly converge on the trigeminocervical nucleus, which was indicated by open lower cervical surgery^[13]^; nucleoplasty resulted in the ablation of nociceptors that innervated the lower cervical disc,^[20,24]^ because nociceptive afferents in the lower cervical disc possibly converge onto the trigeminocervical nucleus through C2–C3 DRG, which was found by an immunohistological analysis of the cervical disc^[25]^; and nucleoplasty possibly produced an indirect effect in improving spinal kinesthetics in the higher cervical spine, which is less likely to be important in mediating headache relief.^[20]^

There are some limitations that need to be acknowledged. First, this study seemly ignored the notion that the upper cervical discs C2–3/C3–4 were also potential sources of the headaches.^[26]^ This was related to the study design. According to the inclusion criteria, all subjects with CEHs were recruited from a group of patients who had undergone nucleoplasty for discogenic or radicular pain in the neck, shoulder, or arm, which mostly originated from lower degenerative cervical discs. Second, the present outcomes derived from this study did not indicate that CEH without discogenic or radicular pain can benefit from cervical nucleoplasty. This needs to be investigated in additional studies. However, compared with discogenic or radicular pain, there are no gold standard diagnostic criteria of CEH for nucleoplasty, which results in difficulties in enrolling subjects.

In summary, this prospective study indicated that CEHs may benefit from nucleoplasty in lower cervical discs in patients with disc herniation accompanied by discogenic or radicular pain in the neck, shoulder, or arm.

## Acknowledgment

The authors thank Shuyue Zheng for help in statistical analyses.
